# A BDS Interference Suppression Technique Based on Linear Phase Adaptive IIR Notch Filters

**DOI:** 10.3390/s18051515

**Published:** 2018-05-11

**Authors:** Hongbo Zhao, Yinan Hu, Hua Sun, Wenquan Feng

**Affiliations:** School of Electronic and Information Engineering, Beihang University, No. 37 Xueyuan Road, Haidian District, Beijing 100191, China; chaichai1994@buaa.edu.cn (Y.H.); sun@buaa.edu.cn (H.S.); buaafwq@buaa.edu.cn (W.F.)

**Keywords:** interference suppression, correlation peak distortion, non-linear phase compensation, adaptive linear phase IIR notch filter

## Abstract

The continuous wave interferences (CWIs) and the narrow-band interferences (NBIs) have significantly impacted the acquisition, tracking and positioning accuracy of Beidou Navigation Satellite System (BDS) receivers. As an interference suppression technology with a simple structure and low hardware cost, the adaptive infinite-duration impulse response (IIR) notch filter has been widely employed in the receivers to mitigate the CWIs and the NBIs. However, the nonlinear phase characteristics introduced by the IIR notch filters into the navigation receivers, may cause the distortion of navigation signals. It also leads to amplitude and phase distortion of the correlation peak in acquisition and loop tracking, which consequently brings on positioning errors in the measurement domain. This problem, however, has been ignored by many previous papers. Meanwhile, some other researchers came up with the idea of equalizers and all-pass networks compensating the distortion, which is also highly infeasible. Therefore, we propose a new method of an adaptive linear phase IIR notch filter with low hardware cost which is composed of three parts—the complex IIR notch filters, the IIR linear phase structure, and the adaptive and variable step-size algorithms. Applying this method, interference suppression and the correlation peak distortion compensation can be achieved with a modest increase hardware cost. This paper compares the performance of the new method with other IIR filters in both CWI and NBI scene and presents the effects of its parameters on the anti-jamming performance. Simulation results show that the proposed module has better anti-jamming performance in NBI scene and can compensate for the correlation peak distortion in the meantime.

## 1. Introduction

China has successfully launched the third-generation satellites of its BDS in the first step of establishing a vast positioning and navigation network with global coverage. However, the radio frequency interference severely influences BDS system on integrity, accuracy, continuity, and availability. Although the spread-spectrum processing gains vary from 30 to 40 dB, high interference signal ratio (ISR) interference with still cause significant positioning errors or paralysis on BDS receivers. Generally, there are several approaches to mitigate the interferences: time domain [1,2,3,4], frequency domain [5,6,7] and space domain [8,9,10].

As a time-domain interference suppression technology with a simple structure and low hardware cost, IIR notch filters have been widely employed in the navigation satellite receivers to mitigate the CWIs and the NBIs. Currently, many authors have paid considerable attention to such method. In [1], the authors used two-pole IIR notch filters to estimate the frequency of interferences and suppress them with another notch-depth adjustable IIR notch filter. Punchalard [3] implemented a modified gradient algorithm to improve the convergence speed of a first-order complex adaptive IIR notch filter. The complex IIR notch filter is mainly employed on Quadrature Phase Shift Keying (QPSK) systems. The coefficients are complex-valued, and the filter can simultaneously mitigate the signals on the in-phase (I) branch and the quadrature (Q) branch. In [2], a single order complex IIR notch filter was also proposed based on a least mean square (LMS) algorithm and a cascade structure to mitigate multi-CWIs. In [11], a new adaptive all-pass based notch filter was employed for narrowband/FM interference suppression and frequency estimation in GPS receivers. However, in its anti-jamming modules, no impact of nonlinear phase characteristic introduced by the IIR notch filters was considered, although such nonlinear phase characteristics may cause the amplitude and phase distortions of the correlation peak in the acquisition and tracking loops, and consequently lead to positioning errors in the measurement domain.

Some researchers have also analyzed the reasons for the distortion caused by the IIR notch filter. In [12], it was concluded that IIR notch filter would bring in a time delay error when it measures the pseudo-range. In [13], the authors used an adaptive linear interpolation filter and linear prediction filter in a direct sequence spread spectrum system and discussed their effects on the correlation peaks. They also indicated that a high side lobe level caused by the nonlinear characteristic could severely impact the acquisition loops. Chien [14] proposed a lattice IIR notch filter and analyzed its effects on the acquisition and tracking loop. Simulation results showed that such problem could be effectively omitted if the −3 dB bandwidth of the adaptive notch filters is adequately controlled.

To eliminate the nonlinear phase, Powell and Chau [15] proposed a linear phase IIR filter structure with last in first out modules (LIFO) and over-lap add modules, but they didn’t apply an adaptive algorithm for interference suppression. In [16], a digital approximate linear phase notch filter was proposed by using two parallel all-pass filters. But the adaptive algorithm still cannot be used in such linear filter. Besides, others brought on some all-pass networks and equalizers to realize the linear phase of the IIR notch filter. In [17], the authors put forward an IIR notch filter with approximately linear phase with two all-pass sub-filters, whereas the computing complexity of coefficients was not low enough.

Considering that BDS B1 signal employs the QPSK modulation, in this paper, we combine the single order complex IIR notch filters and linear phase filter structure to accomplish the linear phase of the IIR notch filter and designed the variable step-size LMS algorithm for adaptively suppressing the interferences. This filter can be employed on BDS receivers to mitigate the CWI and NBI and to make a compensation for the correlation peak distortion.

The following parts of the paper are organized as follows: Section 2 describes the primary signal and system models, in which reasons of correlation peak distortion caused by traditional IIR notch filter are analyzed. Section 3 proposes the linear phase IIR notch filters structure, presenting the design of corresponding adaptive algorithm and variable step-size algorithm are also presented. Section 4 demonstrates the feasibility and availability of proposed method with a comparison between the filter performance of this new filter and others. Finally, the conclusions are drawn in Section 5.

## 2. Signal Models and Distortion Analysis

Before presenting the proposed method, firstly we need to introduce the signal model used in this paper. It is started with the BDS signal and interference model followed by the complex adaptive IIR notch filter. After that, we analyze the reason for the correlation peak distortion in theoretical derivation.

### 2.1. Signal Model

**Figure 1:**
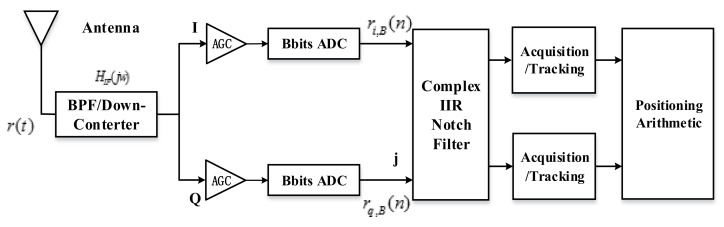
The BDS anti-jamming receiver structure based on complex IIR notch filter.

Figure 1 shows the BDS anti-jamming receiver structures based on the complex IIR notch filters. The BDS B1 signal is composed of I-signal and Q-signal which are in quadrature with each other. It may suffer from CWIs and NBIs intentionally or unintentionally. The signal is received by the receiver antenna, after which it passes the band-pass filter, down converter, automatic gain control (AGC) and analog to digital converter (ADC).

Finally, the sampled navigation intermediate-frequency signals *r_B_*(*n*) are obtained. Its complex expressions are as follows:

(1) $$\begin{array}{cc} r_{B}(n) & =r_{I,B}(n)+jr_{Q,B}(n)\\ & =\sum_{i=1}^{N_{sat}}s^{i}(n)+\sum_{m=1}^{K}J^{m}(n)+N(n) \end{array}$$

where *B* is the quantization bits. *N_sat_* represents the number of the visible navigation satellites. *K* is the number of interferences. *s^i^*(*n*) denotes the *i*-th navigation satellites signals. *J^m^*(*n*) is the *m*-th interference. *N*(*n*) represents the white Gaussian noise with zero mean and one-sided power spectral density *N*_0_. Concretely, the models of the navigation signals are as follows:

(2) $$\begin{array}{c} s^{i}(n)=\sqrt{2P_{I}^{i}}D_{I}^{i}(nT_{s})c_{I}^{i}(nT_{s})\cos[2\pi (f_{L}+f_{dI}^{i})nT_{s}+\theta_{I}^{i}]\\ +j\sqrt{2P_{Q}^{i}}c_{Q}^{i}(nT_{s})\sin[2\pi (f_{L}+f_{dQ}^{i})nT_{s}+\theta_{Q}^{i}] \end{array}$$

where the index *I* represents the in-phase channel. *Q* represents the quadrature channel. $P_{I}^{i}$ denotes the signal power. $D_{I}^{i}\left(nT_{s}\right)$ and $c_{I}^{i}\left(nT_{s}\right)$ represent navigation data and pseudo-random sequence (PRN), respectively. $f_{L}$ and $f_{dI}^{i}$ are the transmitting frequency and Doppler frequency shift, respectively. $\theta_{I}^{i}$ is the carrier phase with uniform distribution. And in the practical conditions, $P_{I}^{i}$ equals to $P_{Q}^{i}$. $f_{dI}^{i}$ equals to $f_{dQ}^{i}$.

The jamming signals can be modeled as follows:

(3) $$J^{m}(n)=\sqrt{2P_{{}^{I}}^{m}}[\cos(2\pi f_{{}^{I}}^{m}nT_{s}+\theta_{{}^{I}}^{m})+j\sin(2\pi f_{{}^{Q}}^{m}nT_{s}+\theta_{Q}^{m})]$$

(4) $$\mathrm{or}\;J^{m}(n)=-\sum_{i=1}^{p}w_{i}J^{m}(n-1)+\varepsilon (n)$$

where Equation (3) is the CWIs model. $P_{I}^{m}$, $f_{I}^{m}$ and $\theta_{I}^{m}$ denote the jam power, frequency, and carrier phase of the CWIs, respectively. Equation (4) represents the Auto-Regression (AR) model. In essence, the output of AR model can be considered as the narrowband interferences.

**Figure 2:**
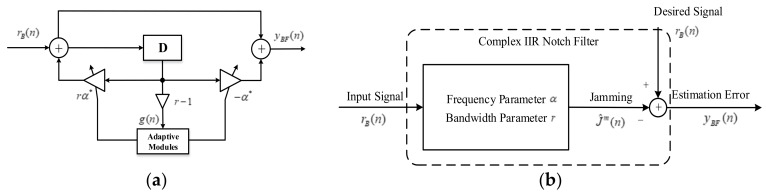
Single order complex adaptive IIR notch filter schematic (**a**) Its normal structure (**b**) Its equivalent Wiener Filter structure.

Figure 2a shows the single order complex adaptive IIR notch filter in the satellite navigation receivers. $r_{B}\left(n\right)$, $y_{BF}\left(n\right)$ and g(n) are the input signals, output signals, and adaptive signals, respectively. D represents the delay module. The complex IIR filter’s system function and its difference equation can be represented as follows:

(5) $$\begin{array}{c} H(z)=\frac{Y(z)}{X(z)}=\frac{1-\alpha^{*}z^{-1}}{1-r\alpha^{*}z^{-1}}\\ y_{BF}(n)=r_{B}(n)-[\alpha^{*}r_{B}(n-1)-r\alpha^{*}y_{BF}(n-1)] \end{array}$$

where X(z) and Y(z) are the *z*-transform of the input and output signals. *r* and α are the notch bandwidth and frequency parameters. They control the −3 dB bandwidth and the notch frequency. The IIR notch filter is stable if the *r* is smaller than 1. They can be written as:

(6) $$\begin{array}{l} BW=(1-r)\pi \\ \alpha =e^{-j\omega } \end{array}$$

where *BW* denotes the −3 dB bandwidth. *ω* is the notch frequency. And the system function of IIR can be given by:

(7) $$h(n)=\sum_{i=\infty }^{\infty }h_{i}\delta (n-i)$$

where $h_{i}$ is the coefficient of the unit impulse response. δ(n) is the Dirac function.

To adjust the notch frequency parameter according to the interference frequency, we employ adaptive algorithms. In the adaptive filter theory [18], Wiener filter is a linear optimal filter based on the minimum mean square error (MSE). It extracts useful signals from stationary noise. In the navigation signal anti-jamming fields, the navigation signals, and white Gauss noise are considered to be the stationary noise. And the narrowband interferences are regarded as the useful signals.

Figure 2b shows the equivalent Wiener Filter structure of Figure 2a. We can get the predicted interference ${\hat{J}}^{m}\left(n\right)$ from the input signal $r_{B}\left(n\right)$. We also regard the received signal $r_{B}\left(n\right)$ as the desired signal in Figure 2b, so the output signal $y_{BF}\left(n\right)=r_{B}\left(n\right)-{\hat{J}}^{m}\left(n\right)$ only contains the navigation signals and white Gauss noise. The adaptive algorithm adjusts its frequency parameters α to make the mean square minimum. The mean square can be express as follows:

(8) $$E[{\left|y_{BF}(n)\right|}^{2}]=E\left({\left|r_{B}(n)-{\hat{J}}^{m}(n)\right|}^{2}\right)=E\{[r_{B}(n)-{\hat{J}}^{m}(n)][r_{B}(n)-{\hat{J}}^{m}(n)]*\}$$

The solution of the frequency parameter is the solution of the Wiener-Hopf equation. However, it needs to calculate the inverse of the matrix which requires a tremendous hardware cost. So, the least mean square (LMS) algorithm has been adopted to simplify the computation. The adaptive algorithm is introduced in Section 3.3.

Without loss of generality, we assume that the interference has been entirely notched after the signals pass the adaptive IIR notch filter. Therefore, we obtain the output signals of filter $y_{BF}\left(n\right)$:

### 2.2. Distortion Analysis

The correlation peak will distort in acquisition and tracking modules. We analysis the distortion reason in this section.

**Figure 3:**
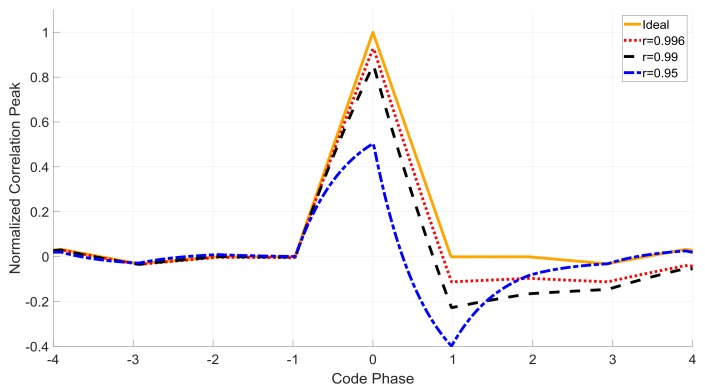
Comparison among different notch bandwidth parameter *r*.

After multiplying $y_{BF}\left(n\right)$ with the local carrier $q\left(n\right)=\exp\left\{-j2\pi \left(f_{IF}+f_{d}\right)nT_{s}\right\}$ and the local PRN sequence $c^{i}\left(mT_{s}-\hat{\tau }\right)$, the signals enter the integrate and dump modules. The output of the prompt correlator $I_{p}$ can be expressed as follows [14]:

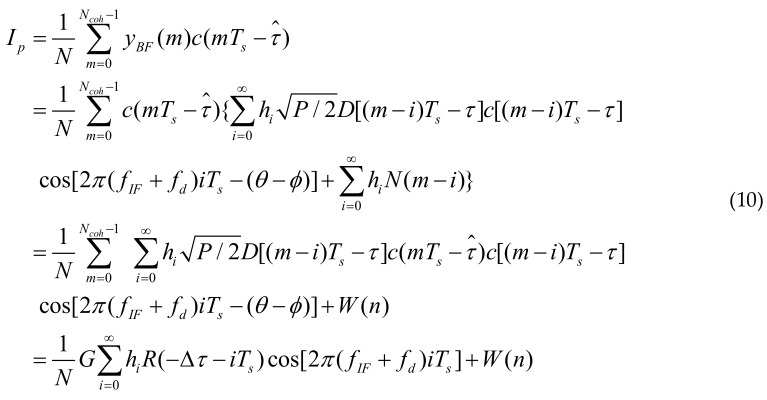
(10)
where *G* is the function of the AGC gain and signal-received power. $\hat{\tau }$ is the time delay estimation. R(τ) is the correlation function. Δτ is the delay time offset. $N_{coh}$ is the sample number of the integrate and dump filter for each integration. W(n) is the white Gaussian noise after processing. Equation (10) indicates that the prompt correlation result $I_{p}$ is the sum of the delayed correlation peaks weighed by the filter coefficients. Figure 3 shows the distorted correlation peaks with different −3 dB notch bandwidth parameters.

Equation (10) shows that the correlation peaks will distort on amplitude and phase compared with the ideal one. There are two reasons for the distortion:

- After the interference suppression, the partial power in the notch bandwidth of navigation signals is lost, which causes the decline of the correlation peak value, yet without affecting the symmetry.
- From Equation (10), the nonlinear phase characteristic of IIR notch filters adds the ideal correlation peaks with the delayed correlation peaks together. And we can see the amplitude and phase distortion on the correlation results from Equation (5),

(11) $$\begin{array}{cc} y_{BF}(n) & =r_{B}(n)-[\alpha^{*}r_{B}(n-1)-r\alpha^{*}y_{BF}(n-1)]\\ & =r_{B}(n)+(r-1)\alpha^{*}r_{B}(n-1)+(r-1)r{\left(\alpha^{*}\right)}^{2}r_{B}(n-2)+\\ & \ldots +(r-1)r^{D-1}(\alpha^{*})Dr_{B}(n-D)+\ldots \\ & =h_{0}r_{B}(n)+h_{1}r_{B}(n-1)+h_{2}r_{B}(n-2)+\ldots +h_{D}r_{B}(n-D)+\ldots \end{array}$$

where, $h_{D}=\left(r-1\right)r^{D-1}{\left(\alpha^{*}\right)}^{D}$ (*D* > 1). Now we consider the absolute value of $h_{D}$. That is due to the effect of absolute values of $h_{D}$ on the correlation amplitude. And from the equation above, the $\left|h_{D}\right|$ increases when *r* decreases, which means the delayed correlation peaks value increases and causes serious distortion. The simulation results in Figure 3 prove that when the parameter *r* decreases, the nonlinear phase characteristic causes more severe distortions.

**Figure 4:**
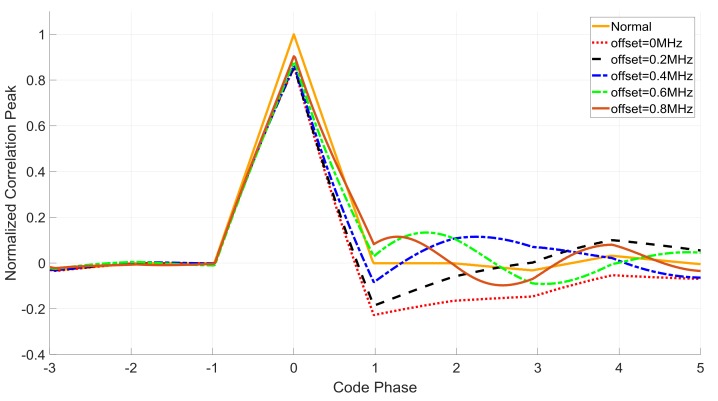
Comparison among different jam frequency offset.

The paper [4] shows that the for C/A code receivers, NBIs at frequencies away from band center degrade code accuracy more than that at band center. To illustrate this, we also compare the correlation peaks with different notch center frequencies—the simulation results shown in Figure 4 indicate that with the different jam offset frequency the distortion degrees also differ from each other.

To describe the distortion degree of the correlation peaks, we bring in the ∆-ratio test [19], which represents the symmetry of the correlation peaks. It can be expressed as Equation (12):

(12) $$\Delta =\frac{\left|I_{e}-I_{l}\right|}{\varepsilon I_{p}}$$

where $I_{e}$, $I_{l}$ and $I_{p}$ represent the coherent integration or non-coherent integration output of early, late, and prompt branches, respectively. *ε* is a constant value related to the correlator spacing. Generally, the correlator spacing between early and late correlator is half of the code chip; and the *ε* equals to 0.5. Under ideal circumstances, the ∆-ratio is zero. However, when the correlation peaks distort, the difference between $I_{e}$ and $I_{l}$ increases; and the value of ∆ also becomes larger. Assuming that the tracking loop has adopted the coherent dot power method in DLL, the formula is as follows [20]:

(13) $$\rho_{cp}=\frac{1}{4}\frac{I_{E}-I_{L}}{I_{P}}$$

where, the $\rho_{cp}$ is the code phase error. Then, the relationship between code phase error and ∆-ratio test is $\rho_{cp}=\Delta \varepsilon /4$. We can, therefore, conclude that in this case, the smaller ∆ is, the smaller the code error is.

## 3. IIR Notch Filter Based on the Linear Phase Structure

In [14], Chien concluded that if the −3 dB bandwidth of the IIR notch filter is less than about 30 KHz, the filters brings almost zero offset on both the acquisition and tracking loops. However, the power amplifiers, up-converters, and other nonlinear components in the jammer widen the CWIs’ bandwidth. And the signal processing, such as signal truncation, may also result in a wider CWIs bandwidth. What’s more, for improving the application range, the −3 dB bandwidth of the IIR filter should not be limited under 30 KHz. Therefore, when the −3 dB bandwidth gets wider, the effects of the nonlinear phase characteristic on the acquisition and tracking loop cannot be neglected.

In this section, we combine the single order complex IIR notch filter with the linear phase IIR structure in [15] to compensate the nonlinear effects. The filters can apply to the BDS receivers to reject the CWIs and NBIs and we also design the adaptive algorithm and variable step-size algorithm to the corresponding linear structure.

### 3.1. Linear Phase IIR Notch Filter Structure

**Figure 5:**
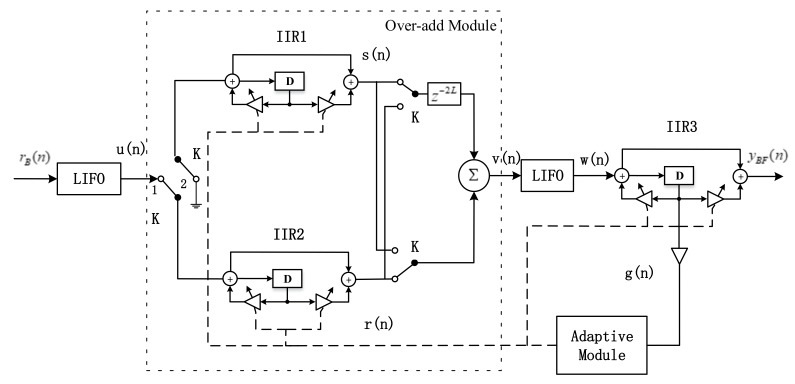
The second order complex linear phase IIR notch filter structure.

**Figure 6:**
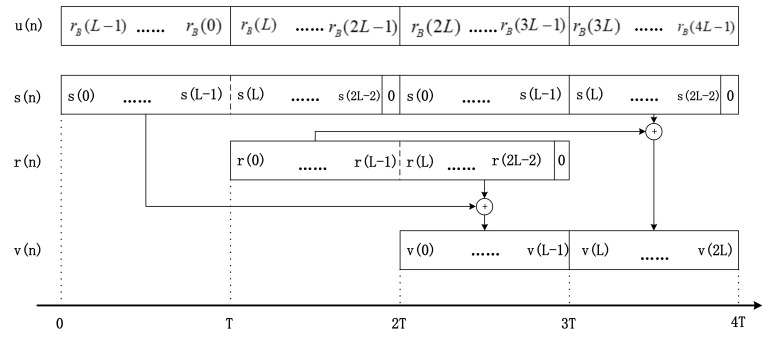
The sequence chart of the overlap-add modules.

In this section, we propose the complex linear phase IIR notch filter, which is based on the linear phase IIR structure, single order complex IIR notch filters, and the corresponding adaptive algorithm and variable-step algorithm. The structure presents as follows (Figure 5):

Figure 5 shows that this module is composed of the LIFO modules, an overlap-add module, and an adaptive module. The LIFO modules are composed of the *L* registers, which can make the *L* signal samples reversed in the time domain. The overlapped modules are including a delay module, an adder, two complex IIR filters and three switches. Its primary task is to realize the sectioned convolution. The input of the two IIR filters is controlled by the switch-*K*, which change its direction every *L* samples. And the output signals are composed of a superposition of the two single order complex IIR notch filters output. Figure 6 shows the signal sequence chart to explain how the overlap-add module works to achieve the sectioned convolution.

Its theoretical analysis is demonstrated as follows. The input of the sectioned convolution can be expressed as follows:

(14) $$\begin{array}{c} u(n)=\sum_{k=0}^{\infty }r_{B,k}(n)\\ r_{B,k}(n)=\left\{ \begin{array}{l} r_{B}(n),kL\leq n\leq (k+1)L-1\\ 0,otherwise \end{array}\right. \end{array}$$

The output signal v(n) can be expressed as:

(15) $$\begin{array}{c} v(n)=\sum_{k=0}^{\infty }v_{k}(n)\\ where,v_{k}(n)=\left\{ \begin{array}{l} v(n),kL\leq n\leq (k+1)L-1\\ 0,otherwise \end{array}\right. \end{array}$$

and the $v_{k}\left(n\right)$ can be shown by:

(16) $$\begin{array}{cc} v_{k}(n) & =\sum_{m=n-L}^{n}u(m)h^{*}{}_{n-m}\\ & =\sum_{m=n-L}^{kL-1}u(m)h^{*}{}_{n-m}+\sum_{m=kL}^{n}u(m)h^{*}{}_{n-m} \end{array}$$

The *k*-th part and the (*k* + 1)-th part of the convolution result can be expressed as follows:

(17) $$u_{k}(n)*h(n)=\sum_{m=(k-1)L}^{kL-1}u(m)h^{*}{}_{n-m}=\sum_{m=(k-1)L}^{n-L-1}u(m)h^{*}{}_{n-m}+\sum_{m=n-L}^{kL-1}u(m)h^{*}{}_{n-m}$$

(18) $$u_{k+1}(n)*h(n)=\sum_{m=kL}^{(k+1)L-1}u(m)h^{*}{}_{n-m}=\sum_{m=kL}^{n}u(m)h^{*}{}_{n-m}+\sum_{m=n+1}^{(k+1)L-1}u(m)h^{*}{}_{n-m}$$

We can see that the convolution results v(n) in Equation (16) are composed of two parts. The first part is from the second item of the Equation (17), and the second part is from the Equation (18)’s first item. In summary, the convolution results of the whole sequence can be calculated by the sum of the sectioned convolution results.

And according to the adaptive algorithm mentioned below, the input signal of the adaptive modules are the gradient signal of the output signal of the IIR filter, which can be represented as Equation (24). And the output of the adaptive modules adjusts the notch frequency parameter *r* and the step parameter *μ* of the three notch filters simultaneously. In summary, the signal processing procedure is as follows:

- Step 1: The navigation signals enter the LIFO modules to make the signal time reversed, which indicates u(n)=x(−n);
- Step 2: u(n) passes the overlap-add modules for sectioned convolution;
- Step 3: The signal v(n) passes the second LIFO modules. After that, the input signals x(n) are equivalent to pass a non-causal filter, whose unit impulse response is expressed as h(−n);
- Step 4: The IIR3 outputs the final signals which is also the output of the linear phase IIR notch filter.
- Step 5: And consider the output signal y(n) and the adaptive signals g(n) of the last level notch filter as the adaptive modules input. It adjusts the notch frequency and −3 dB bandwidth parameters.

We prove that the proposed filter has a linear phase characteristic from the time domain and frequency domain. In the time domain, the proposed modules, in essence, are composed of two cascaded single order notch filters. There unit impulse responses are h(−n) and h(n), respectively. The cascade of the two filters constitutes a second order linear phase IIR notch filter. Its unit impulse response is an even symmetry sequence. The system function can be expressed as: $h^{\prime }\left(n\right)=h\left(-n\right)*h\left(n\right)$. Its coefficients are even-symmetric. The symbol ∗ represents the convolution. The sufficient and necessary conditions for the filter’s linear phase characteristic is that the coefficients of the time-domain system function are even or odd sequence. As a conclusion, the proposed structure in Figure 6 has the linear phase characteristic.

From the frequency domain, the proofs are expressed as follows:

(19) $$U(z)=X(z^{-1})$$

(20) $$V(z)=U(z)H(z)=X(z^{-1})H(z)$$

(21) $$W(z)=V(z^{-1})=X(z)H(z^{-1})$$

(22) $$Y(z)=W(z)H(z)=X(z){\left|H(z)\right|}^{2}$$

where U(z), V(z) and W(z) are the Z-transform of the u(n), v(n) and w(n), respectively in Figure 5. Equation (22) shows that the output signals are relevant to the magnitude characteristic of the IIR filter without non-linear phase characteristic. Therefore, this structure can be employed to mitigate the correlation peaks distortion caused by the nonlinear phase characteristic. In essence, the proposed filter is a second order IIR filter. It has almost the equal anti-jamming performance comparing with the other traditional notch filters.

Generally, in some cases which need strict linear phase, such as high-accuracy survey and mapping, the all-pass networks and equalizers will be added to compensate the nonlinear phase effect. But the design of all-pass network and equalizer is more challenging to design an IIR notch filter. The structure of the linear phase IIR notch filter only needs three single order complex IIR filters to meet the linear phase characteristic needs.

The system complexity and the hardware cost of the proposed filter are discussed as follows. To simplify the calculation, we adopt the following numbers to calculate for every *L* samples. Concerning system complexity, samples in IIR notch filter need 2*L* multiplication and 2*L* addition. *L* additions are needed in over-add modules. 4*L* addition, 4*L* + 3 multiplication, and 2 subtractions are also needed in adaptive algorithm modules, so the proposed filter’s system complexity is *O*(14*L* + 5), that is *O*(*L*). Regarding the hardware cost, only 6*L* + 3 registers, 2*L* + 7 summing units, 2*L* + 10 multiplying units and two dividers are needed to meet the filter’s need. Consequently, both system complexity and hardware cost are low enough for use in low-cost receivers.

### 3.2. Corresponding Adaptive and Variable Step-Size Algorithm

Figure 5 shows that the linear phase structure adapted a block processing method to realize the time-reversal and sectioned-convolution. To maintain the system coherence, we rewrite the adaptive algorithm [14] to the block form. The adaptive algorithm adjusts the α every *K* samples. *K* is the block length. The adaptive algorithm formulations are inferred as follows, it’s the *N*-th renewal process:

(23) $$Y_{BF}(N)=\left[\begin{array}{c} \begin{array}{c} y_{BF}(NK)\\ \begin{array}{c} y_{BF}(NK-1)\\ \ldots \end{array} \end{array}\\ y_{BF}[(N-1)K+1] \end{array}\right]=\left[\begin{array}{c} \begin{array}{c} w(NK)-\alpha^{*}w(NK-1)+r\alpha^{*}y_{BF}(NK-1)\\ \begin{array}{c} w(NK-1)-\alpha^{*}w(NK-2)+r\alpha^{*}y_{BF}(NK-2)\\ \ldots \end{array} \end{array}\\ w[(N-1)K+1]-\alpha^{*}w(NK-K)+r\alpha^{*}y_{BF}(NK-K) \end{array}\right]$$

where $Y_{BF}\left(N\right)$ represents the output signals column vectors with length *K*. w(NK) is the input signal of the IIR3 filter in Figure 5. $Y_{BF}(N)$’s derivative functions for α are expressed as follows. It is regarded as the adaptive signals:

(24) $$\begin{array}{cc} G(N)=\frac{\partial Y_{BF}(N)}{\partial \alpha } & =\left[\begin{array}{c} \begin{array}{c} \begin{array}{c} \frac{\partial y_{BF}(NK)}{\partial \alpha }\\ \frac{\partial y_{BF}(NK-1)}{\partial \alpha } \end{array}\\ \ldots \end{array}\\ \frac{\partial y_{BF}[(N-1)K+1]}{\partial \alpha } \end{array}\right]\\ & =\left[\begin{array}{c} \begin{array}{c} \begin{array}{c} g(NK)\\ g(NK-1) \end{array}\\ \ldots \end{array}\\ g[(N-1)K+1] \end{array}\right]=\left[\begin{array}{c} \begin{array}{c} \begin{array}{c} -2w(NK-1)+2ry_{BF}(NK-1)\\ -2w(NK-2)+2ry_{BF}(NK-2) \end{array}\\ \ldots \end{array}\\ -2w[(N-1)K]+2ry_{BF}[(N-1)K] \end{array}\right] \end{array}$$

According to the LMS algorithm, the instantaneous gradient can be expressed as follows:

(25) $$\begin{array}{cc} \nabla (N) & =\frac{\partial {\left|Y_{BF}(N)\right|}^{2}}{\partial \alpha }=\frac{\partial \left|Y_{BF}^{H}(N)Y_{BF}(N)\right|}{\partial \alpha }\\ & =\frac{\partial {\left|y_{BF}(NK)\right|}^{2}}{\partial \alpha }+\frac{\partial {\left|y_{BF}(NK-1)\right|}^{2}}{\partial \alpha }\ldots +\frac{\partial {\left|y_{BF}[(N-1)K+1]\right|}^{2}}{\partial \alpha }\\ & =y_{BF}^{*}(NK)g(NK)+y_{BF}^{*}(NK-1)g(NK-1)\ldots +y_{BF}^{*}[(N-1)K+1]g[(N-1)K+1]\\ & =Y_{BF}^{H}(N)G(N) \end{array}$$

the iteration formula of the parameter α is as follows:

(26) $$\alpha (N+1)=\alpha (N)-\mu \nabla (N)=\alpha (N)-\mu Y_{BF}^{H}(N)G(N)/K$$

where the superscript *H* represents the complex conjugate transpose. *μ* represents the update step-size. Considering G(N) and $Y_{BF}\left(N\right)$ as the input of the adaptive modules, the filters updated α every *K* samples. Figure 5 shows that the three IIR notch filters need the same system function when handling the same block signals. This linear phase structure cannot make the system function ultimately the same. But the simulation results indicate that if the step parameter *μ* is small enough, the effects on the filter performance can be ignored.

To accelerate the convergence rate, we employ the variable step-size algorithm. The Equation (26) is rewritten as follows:

(27) $$\alpha (N+1)=\alpha (N)-\mu (N)\nabla (N)=\alpha (N)-\mu (N)Y_{BF}^{H}(N)G(N)/K$$

(28) $$\begin{array}{l} \mu (N)=\frac{s}{\varphi (N)}\\ \varphi (N)=\rho \varphi (N-1)+(1-\rho )G^{H}(N)G(N) \end{array}$$

where *s* is the constant step-size for adjusting the convergence rate. φ(N) is an instantaneous power estimation of G(N), whose initial value affects the initial value of the first step-size. And ρ (0<ρ<1) is the forgetting factor which presents the correlation between power estimation of G(N) and G(N−1). After the iteration, the α(N) convergence to a fixed value. That means the filter has entered the steady state. What we are concerned about in steady state is the mean square error (the steady state error) shown in Equation (29). It affects the convergence performance and the stability of the filter:

(29) $$E\left({\left|e(N)\right|}^{2}\right)=E\left(\left|Y_{BF}(N)Y_{BF}{\left(N\right)}^{H}\right|\right)/K$$

### 3.3. Analysis of Filter Performance

As mentioned above, the block length *L*, the notch bandwidth parameter *r* and the block length *K* of the adaptive module are the main factors which affect the performance of the IIR notch filter. In this section, therefore, they are examined in detail.

The block length *L* the number of the LIFO registers, which means the input signals are divided into *L* samples per block. It affects the over-added performance on approximating to the ideal transfer function. The more samples per block, the better each section’s transfer function approximates *H*(*z*). We assume that *L* is larger than impulse response length of the physical realization. Then when  |i|>L, the notch filter coefficient $h_{i}$ is small enough to be neglected.

The −3 dB notch bandwidth parameter *r* can significantly affect the correlation peaks’ symmetry in the traditional IIR filters. But it does little influence on it in the proposed IIR structure. It mainly changes the value of the correlation peaks. Two reasons mainly cause the distortion of the correlation peaks: the power loss of the normal signals and the linear characteristic. As a result, when we need to increase the −3 dB bandwidth of the filter, the power loss also increases, leading to the decrease of the peaks value.

The block length *K* of the adaptive modules also affect the filter performance in the aspects of convergence rate and steady-state error. With the block length increasing and without the variable step-size algorithm, the filter gives a slower convergence rate but less steady-state errors. However, while applying the variable step-size algorithm, the filter converges to smaller steady-state errors than the fixed step-size. Therefore, to guarantee no conflict between the update of the filter coefficients and the sectioned convolution, the relationship between block length *K* and LIFO length *L* should be as follows:

(30) K=i*L (i=1,2…n)

but the simulation shows that K=L is the best choice. The larger *K* is, the more complex the IIR notch filter is.

## 4. Simulation and Verification

**Figure 7:**
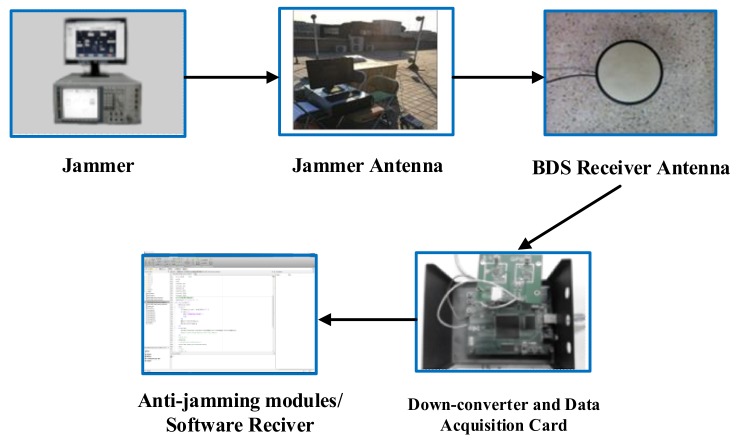
BDS anti-jamming simulation and verification platform.

This section divides into two parts: one is comparing the proposed filter with other filters under CWIs and NBIs scene, respectively; the other is comparing their performance with different parameters. First, the simulation conditions present as follows. Figure 7 demonstrates the BDS anti-jamming simulation and verification platform, which is composed of jammers, BDS signal acquisition system, anti-jamming modules, and software receiver.

Table 1 lists the verification conditions on the system parameters.

### 4.1. Comparison with Other Filters

**Figure 8:**
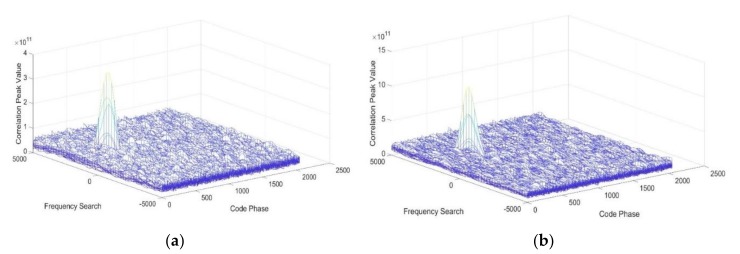

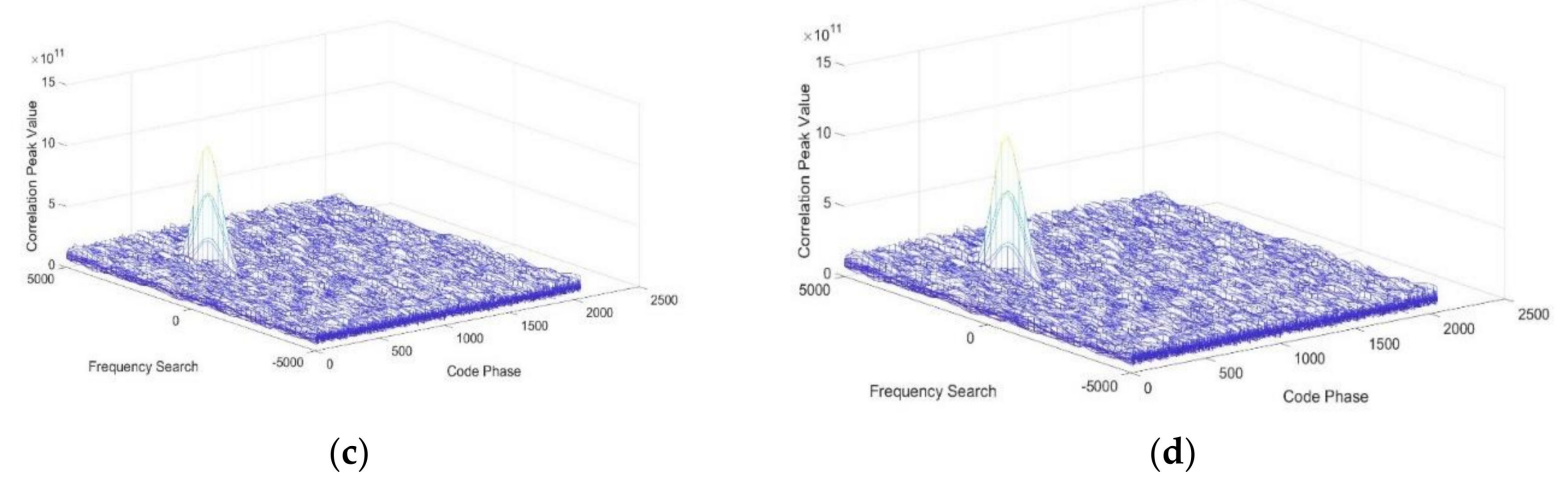
Comparison of the acquisition results under the CWI. (**a**) The acquisition chart results of the Frequency Excision (**b**) The acquisition chart results of Adaptive IIR notch filter (**c**) The acquisition chart results of Chien’s filter (**d**) The acquisition chart results of Proposed Method.

By applying different filters, including adaptive IIR notch filter with LMS algorithm [1], frequency excision method [21], Chien’s filter [14] and the proposed filter, we compare their anti-jamming capabilities and the correlation peak symmetry and set up two application scenarios. One is that the BDS receiver suffers from the CWI, where we set the −3 dB bandwidth of the filters to the same and narrow level. The other is suffering from the NBI, where we also set the −3 dB bandwidth to the same value but a wider level. Figure 8 shows the acquisition chart results of the four filters in the CWI scenario.

**Figure 9:**
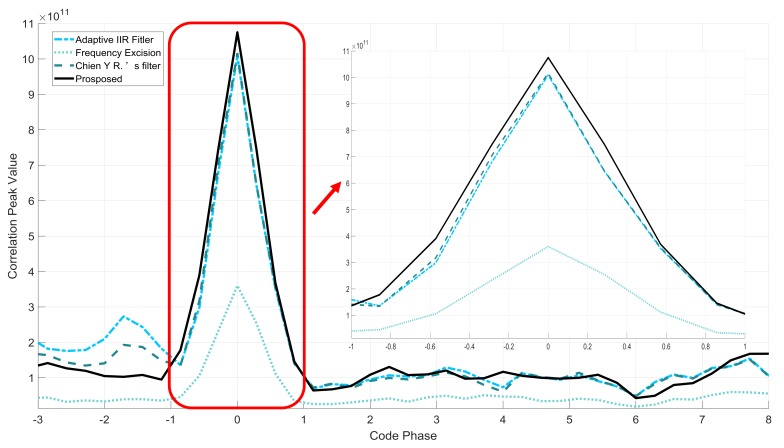
Comparison of the correlation peaks under CWI scene.

**Figure 10:**
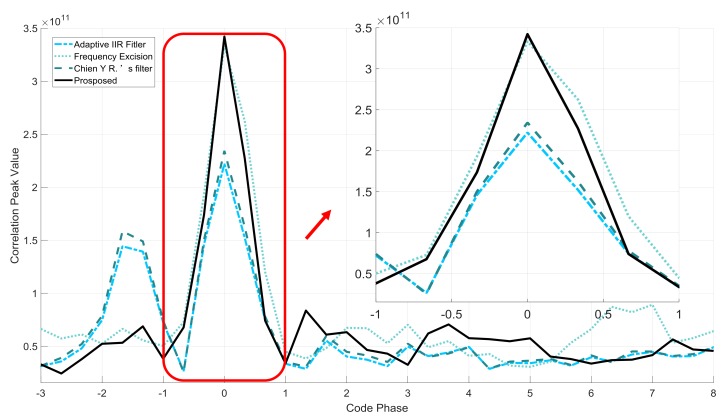
Comparison of the correlation peaks under NBI scene.

The correlation peak values and ∆ ratio test results are listed in Table 2:

From Table 2 and Figure 8, the results show that in the CWI scene, the correlation peak values are almost the same except for the frequency excision. The ∆ ratio tests also have low values. The proposed filter has the same performance in this scenario. However, in the NBI scenario, we can find that all the peak values decrease. Such a phenomenon occurs because the filter mitigates more navigation signal with wider −3 dB bandwidth. Compared with other two IIR filters, the proposed filter has higher correlation peak and lower ∆ ratio value. In the given verification conditions, from the $\;\rho_{cp}$ results, the proposed method reduces the code phase error from 22.11 m to 0.16 m without any other errors. In conclusion, the proposed filter is found to have better interference suppression performance and effectively mitigates the correlation peak distortion. Figure 9 and Figure 10 illustrate the correlation peaks of the four filters with CWI and NBI, respectively.

The frequency excision has almost equal values on the correlation peaks and ∆ ratio value in different scenarios. Figure 9 shows that the proposed filter has the same anti-jamming performance as other filters. Figure 10 shows that in NBI scene, the adaptive IIR notch filter and Chien’s filter could have high side-lobes alongside the main peak without linear characteristics. The nonlinear phase characteristic also causes the asymmetry of the correlation peak. However, the proposed filter has little effects on the correlation peaks.

In conclusion, in the CWI scenario, the IIR notch filters have almost the same anti-jamming performance. However, in the NBI scene, the proposed filter performs better because of its distortionless correlation peak and lower side-lobes.

### 4.2. Anti-jamming Performance with Different Filter Parameters

**Figure 11:**
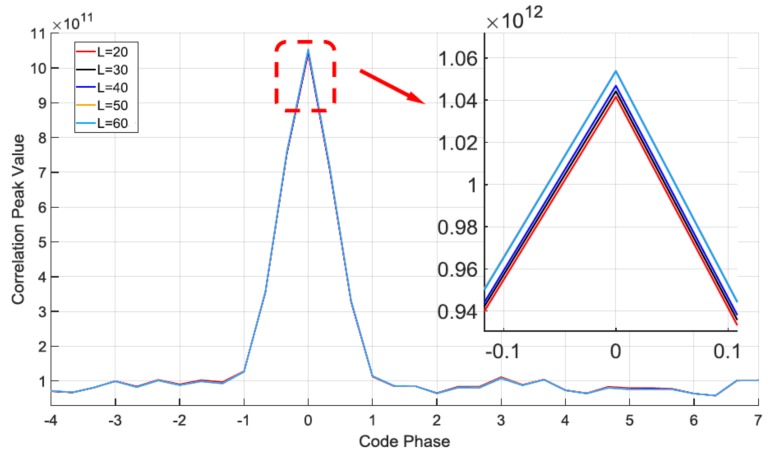
Comparison of the acquisition result with different L.

Besides comparing the correlation peak value and the convergence performance, respectively, we also analyze how the block length *L* and the −3 dB bandwidth parameter *r* affect the proposed filter performance itself. Figure 11 shows the influence the block length *L* has on the correlation peak values:

The result shows the correlation peak values produce nearly the same results with different block length *L*. When *L* is 60, the correlation peak value is 1.052 × 10^12^, and when *L* is 20, the correlation peak is 1.041 × 10^12^. The reason (for what) is that when the length *L* rises to a particular value, the second term on the right side of the Equation (26), $Y^{H}\left(N\right)G\left(N\right)/L$ is equal to the same constant value. In each iteration, we can get $\alpha_{L=20}\left(n\right)=\alpha_{L=30}\left(n\right)=\ldots =\alpha_{L=60}\left(n\right)$. That means the filters with different *L* converge to the same notch frequency in the steady state. With the same −3 dB notch bandwidth and the same notch frequency in the steady state, the block length *L* doesn’t significantly affect the correlation values.

**Figure 12:**
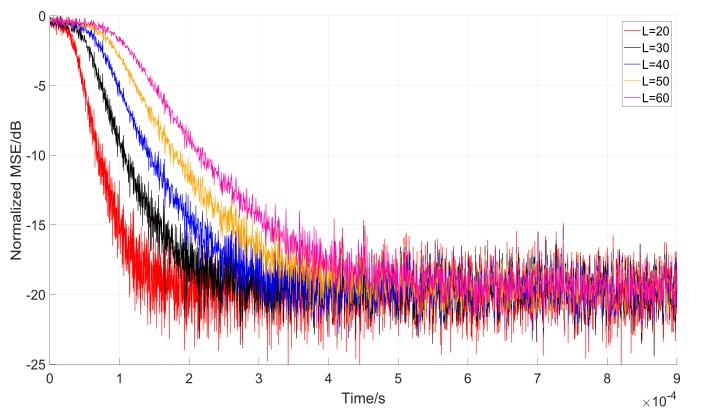
Comparison convergence rate with different block length *L*.

**Figure 13:**
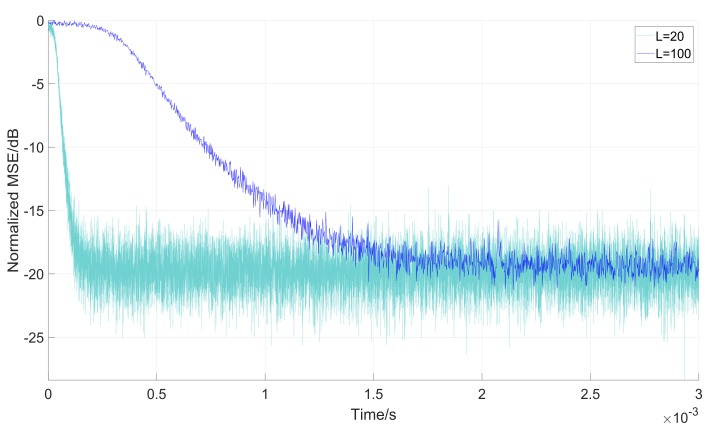
Comparison convergence performance with *L* = 20 and *L* = 100.

Whats’ more, Equation (27) shows that the length *K* (*K* = *L*) affects the convergence rate of parameter α(n), due to which, we analyze the convergence rate with the same variable step-parameter μ(n). Figure 12 and Figure 13 show the convergence performance under the same initial conditions of the parameter α(n) and μ(n).

Figure 12 indicates that with the increasing of parameter *L*, it costs more time to make the notch frequency parameter convergence close to steady-state. The fastest convergence rate is 0.2 ms as the *L* equals to 20. It is three times faster than it is when *L* equals to 60. The block length 60 is also three times larger than *L* = 20. On the premise that the notch frequency parameter α(n) and step parameter μ(n) start from identical initial states. We, therefore, conclude that from each iteration, we can get that $\alpha_{L=20}\left(n\right)=\alpha_{L=30}\left(n\right)=\ldots =\alpha_{L=60}\left(n\right)$. However, the iteration time has a linear relation with block length, so on the timescale, the convergence rate is slower with the increasing length *L*.

While Figure 12 indicates the relationship between convergence rate and the block length *L*. Figure 13 shows the stability of the filter. It can be noticed that the Normalized MSE (*L* = 100) has more unstable steady state errors because of its dependence on the variable step-size. Equation (27) and Equation (28) show that the μ(n) has the same value under the same G(n). Although the convergence rate becomes higher with the increase of *L*, the stability of the steady-state error decreases, which means a more stable state of the filter. As a result, when block length *L* is given, both the convergence rate and the stability of the filter should be taken into consideration.

**Figure 14:**
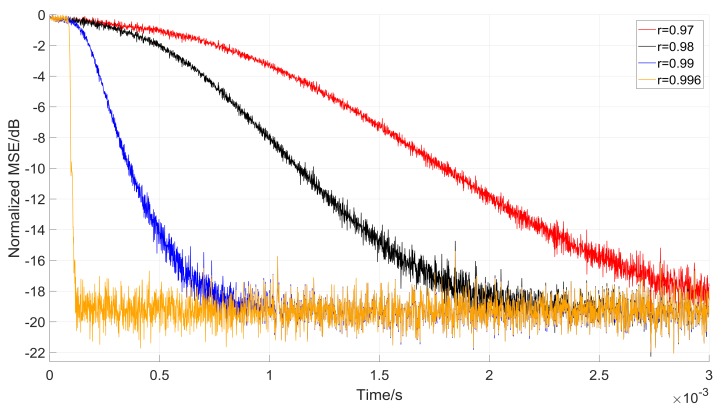
Comparison between different −3 dB bandwidth with *L* = 100.

Meanwhile, we also compare the convergence rate with different parameter *r* and present the results in Figure 14. The presented results indicate that the narrower the −3 dB bandwidth is, the faster the convergence rate is. This can be explained by the fact that when the filters do not notch the interferences, the φ(n) in Equation (28) remains the same—they have the same step-size to converge to steady-state. However, the interferences are firstly notched by the wider −3 dB bandwidth filter; and consequently, the MSE decreases, which makes the step-size smaller and the filter gets a slower convergence rate. Also, we can conclude that the convergence rate is sensitive to the parameter *r*.

## 5. Conclusions

This paper mainly focuses on the non-linear phase characteristics caused by the traditional IIR notch filter after interference suppression and proposes an adaptive linear phase second order IIR notch filter with a small hardware cost. Meanwhile, the corresponding variable step-size adaptive algorithm is designed to improve the convergence performance, which can help the BDS anti-jamming receivers to reject CWIs and NBIs. According to the simulation results, the proposed filter presents almost equal anti-jamming abilities in the CWI scenario compared with other methods, including the adaptive IIR notch filter, frequency excision, as well as Chien’s filter. Contrastively, it performs better on anti-jamming in the NBI scenario, with the capability of mitigating the acquisition distortion and side-lobes at the same time. The block length *L* and the −3 dB bandwidth affect the performance as well. Besides, we also analyze their impacts on filter performance: As *L* decreases, the correlation peak values increase slightly; the convergence rate speeds up; the hardware cost reduces. Besides, the convergence rate also increases with narrower bandwidth. However, the simulation results also indicate that the length *L* should be larger than a certain value, or the filter cannot work normally. Therefore, when designing the linear phase IIR notch filters, researchers should consider the block length *L* and the −3 dB bandwidth as well.

## Figures and Tables

**Table 1:** Simulation Parameters.

Navigation System	BDS B1	
Sample Rate	100 MHz	
Intermediate Frequency	40.098 MHz	
Interference	40.098 MHz CWI	40.098 MHz NBI with 10 KHz bandwidth
Interference Power	−75 dBm	

**Table 2:** Comparison of different interferences on anti-jamming capabilities.

Anti-Jamming Methods	CWI			NBI		
	Correlation Peak Value	∆ Ratio Test	$\rho_{cp}$/Chip	Correlation Peak Value	∆ Ratio Test	$\rho_{cp}$/Chip
**Frequency Excision**	3.518 × 10^11^	0.0119	0.06	3.422 × 10^11^	0.0708	0.0354
**Adaptive IIR notch filter**	1.009 × 10^12^	0.0227	0.0113	2.221 × 10^11^	0.2736	0.1368
**Chien’s filter**	1.017 × 10^12^	0.0295	0.015	2.343 × 10^11^	0.2947	0.1474
**Proposed**	1.075 × 10^12^	**0.0005**	**0.00025**	3.335 × 10^11^	**0.0093**	**0.0046**
